# Supplementary material for: Single-cell RNA sequencing reveals midbrain dopamine neuron diversity emerging during mouse brain development
Source: Nat Commun. 2019 Feb 4;10:581. doi: 10.1038/s41467-019-08453-1 (PMC6362095; doi:10.1038/s41467-019-08453-1)
Supplement: Supplementary file 1 — Supplementary Information [file 41467_2019_8453_MOESM1_ESM.pdf]

Supplementary Information

Tiklová et al., **Single-cell RNA Sequencing Reveals Midbrain Dopamine Neuron Diversity Emerging During Mouse Brain Development**

Supplementary Figures 1-13
Supplementary Tables 1 and 2

Sup.Figure 1

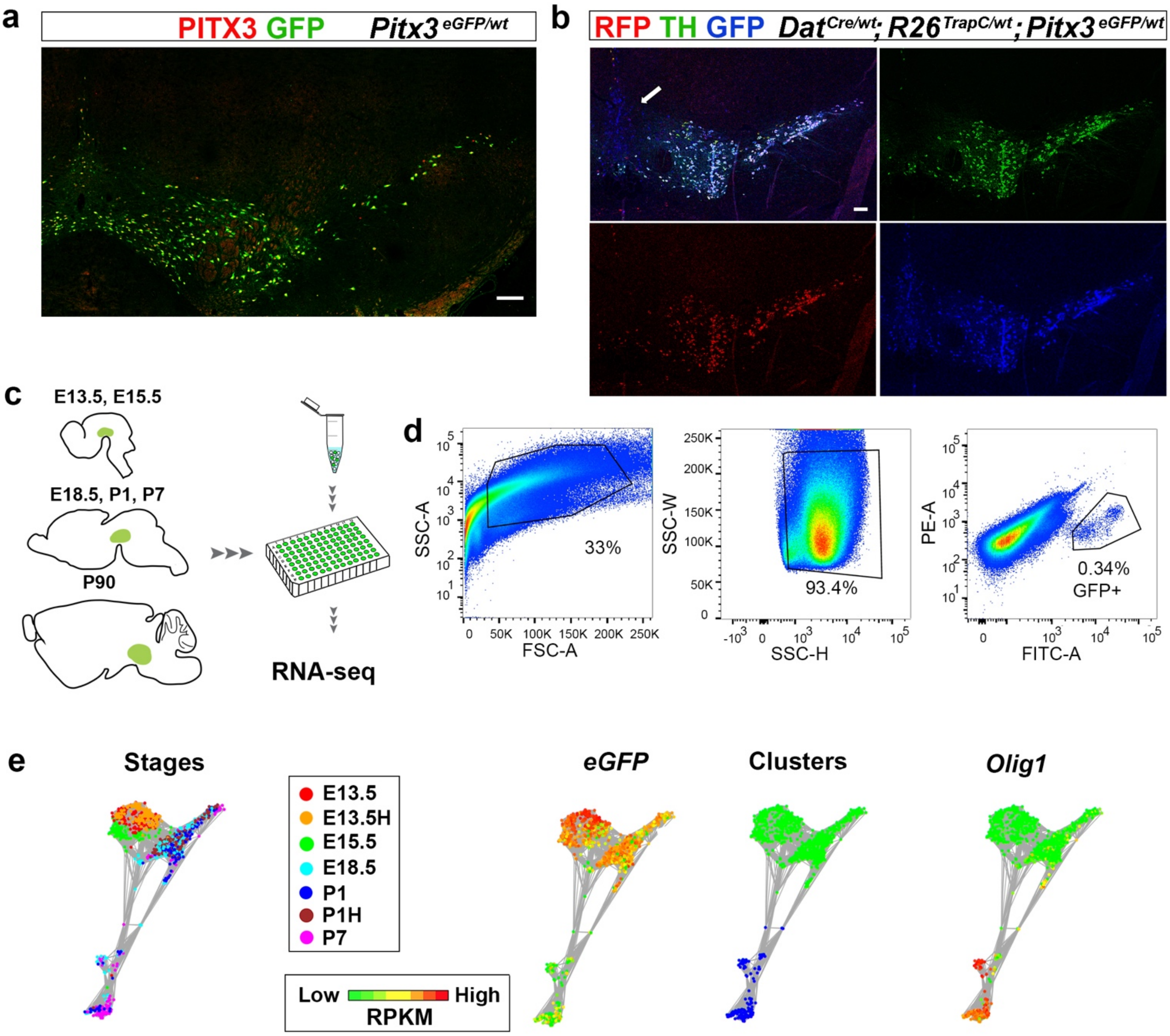

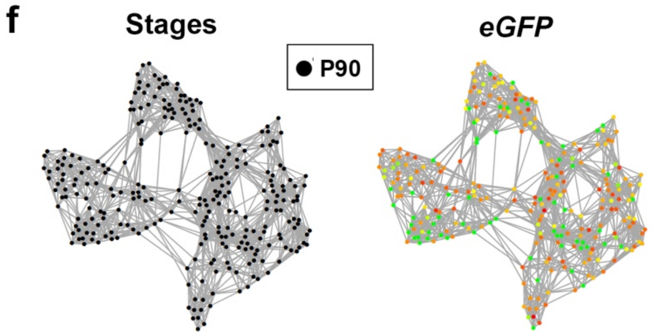

g

Develop. Stage	Number of Cells
E13.5	135
E15.5	140
E18.5	181
P1	258
P7	80
P90	312
E13.5H	141
P1H	148

Supplementary Figure 1. Isolation and sorting of the *Pitx3* single cells
a) Immunostaining analysis of PITX3 and GFP in *Pitx3*$^{eGFP/wt}$ adult mouse brain on a frozen section. Scalebar is 100 µm. b) Immunostaining analysis of RFP, TH and GFP in *Dat*$^{Cre/wt}$*, R26*$^{TrapC/wt}$*, Pitx3*$^{eGFP/wt}$ adult mouse brain on a paraffin section. Arrow points to cells expressing Pitx3-eGFP, but not labelled by Dat-Cre and lacking TH. Scalebar is 100 µm. c) Schematic representation of isolation and scRNA-seq of *Pitx3*$^{eGFP/wt}$ and *Pitx3*$^{eGFP/eGFP}$ single cells from different developmental stages (E13.5, E15.5, E18.5, P1, P7, P90). d) FACS strategy for sorting the single cells from *Pitx3*$^{eGFP/wt}$ and *Pitx3*$^{eGFP/eGFP}$ mice. e) Filtering of non-*eGFP* expressing cells in embryonic and perinatal cells. Network with all cells showing one clear cluster (in blue) with low expression of *eGFP* and high expression of *Olig1*. The cells from this cluster were removed in all subsequent analysis.f) Same type of network as in e) for adult cells. No clear group of non-*eGFP* expressing cells was evident.
g) Table representing the number of single cells from *Pitx3*$^{eGFP/wt}$ and *Pitx3*$^{eGFP/eGFP}$ mice (mutants denoted with an H in the table) from different developmental stages. These cells passed the QC and were included for the further analyses.

Sup.Figure 2

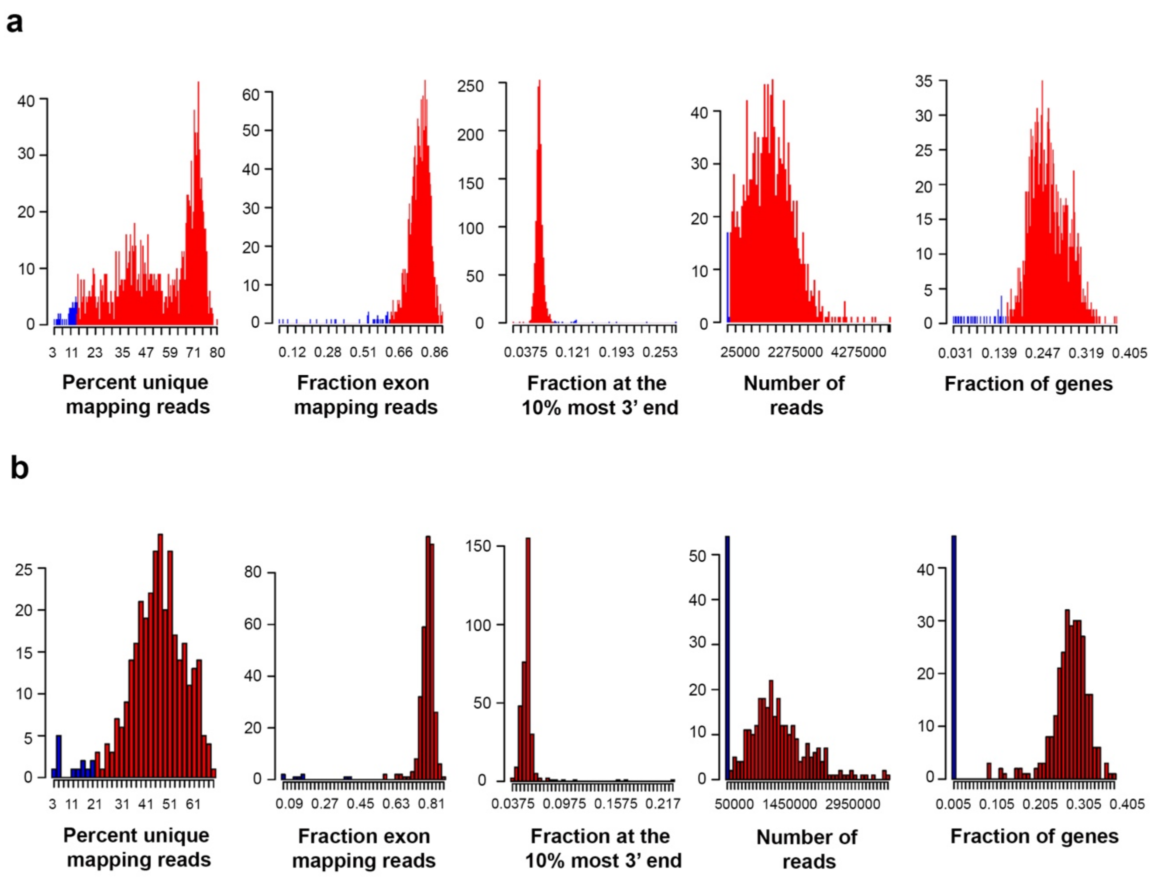

Supplementary Figure 2. RNA-seq Quality Control
a) The quality control of the single cells from E13.5, E15.5, E18.5, P1, P7. The blue color indicates cells which were filtered out.
b) The quality control of the single cells from P90. The blue color indicates cells which were filtered out.
The Y-axis in all of the plots depicts the number of cells.

Sup.Figure 3

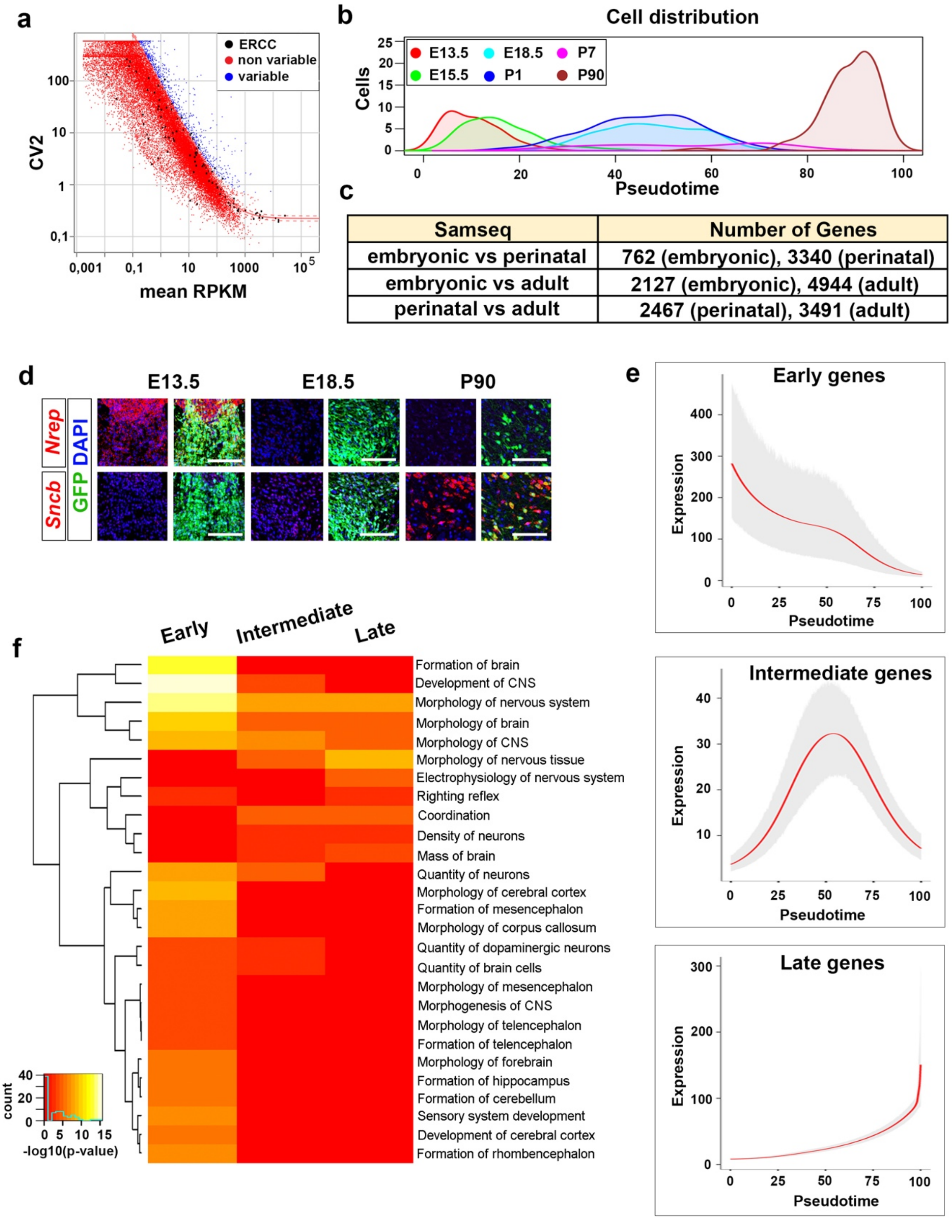

Supplementary Figure 3. Temporal changes in gene expression during postmitotic maturation of *Pitx3*-expressing cells

a) Identification of highly variable genes across the cells. Technical noise fit: squared coefficients of variation are plotted against the means of RPKM.

b) Distribution of analyzed cells along the pseudotime, colored by developmental stage.

c) Table representing number of significantly changed genes between different maturation stages.

d) Fluorescent ISH of *Sncb* and *Nrep* genes at different developmental stages on paraffin sections. PITX3 expression was detected by GFP staining of the *Pitx3*$^{eGFP/wt}$ mouse. Scalebars are 100 µm.

e) Expression profile of gene clusters along pseudotime.

f) Selected functional categories from IPA analysis of the gene clusters in 2e.

Sup.Figure 4

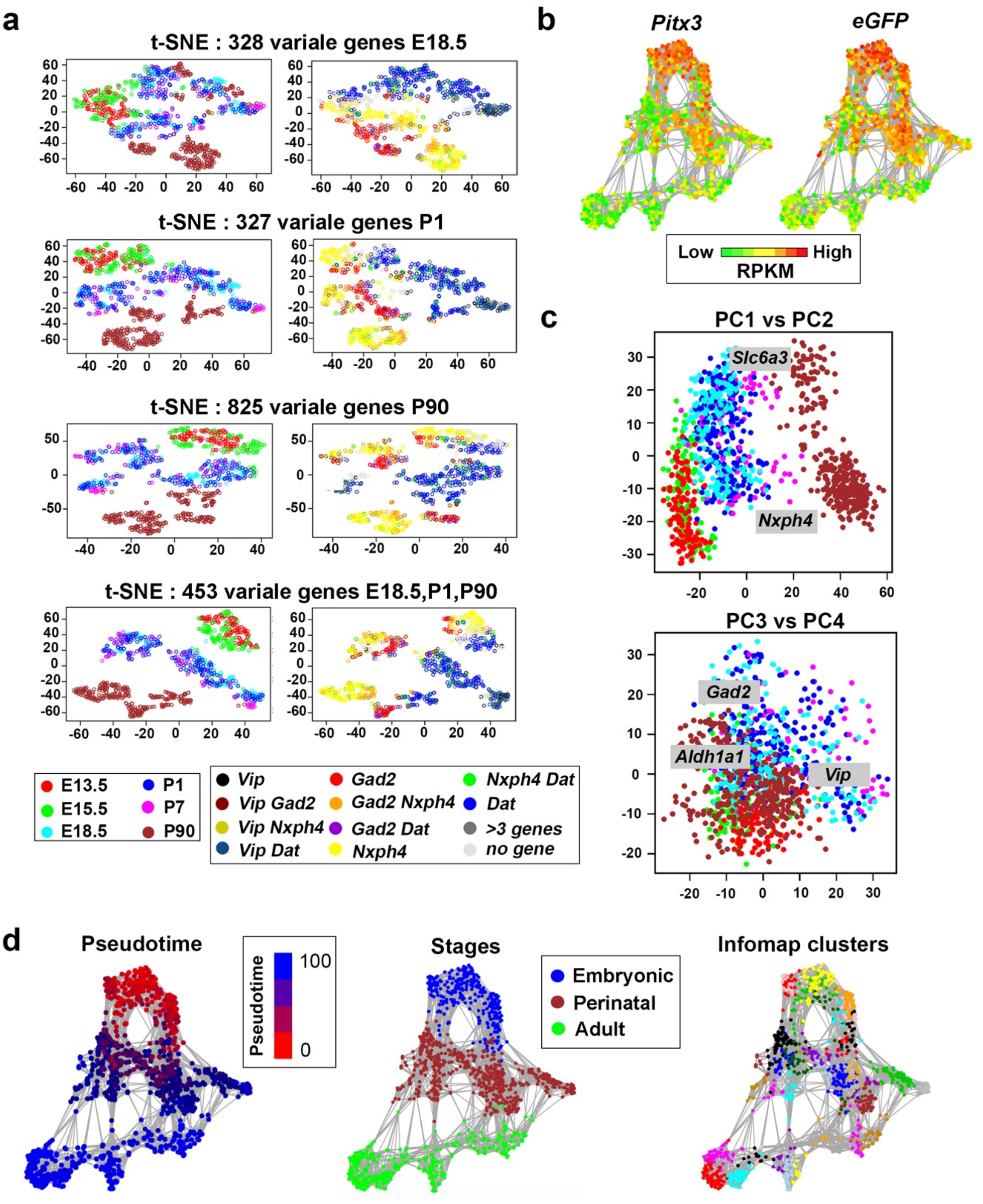

Supplementary Figure 4. t-SNE network and definition of the subgroups

a) Four t-SNE runs with different sets of variable genes, colored by developmental stage (left) and by expression of main celltype marker genes with RPKM>150 (right).

b) The expression of *Pitx3* and *eGFP* genes visualized on the network. The *Pitx3* and *eGFP* resemble the same pattern of expression.

c) PCA plots demonstrating the distribution of the $Pitx3^{eGFP/wt}$ single cells. The top loading genes from the corresponding PCA are highlighted.

d) Network plot colored by pseudotime or developmental stage. Network was divided into 48 clusters with unsupervised clustering done with Infomap community detection.

Sup. Figure 5

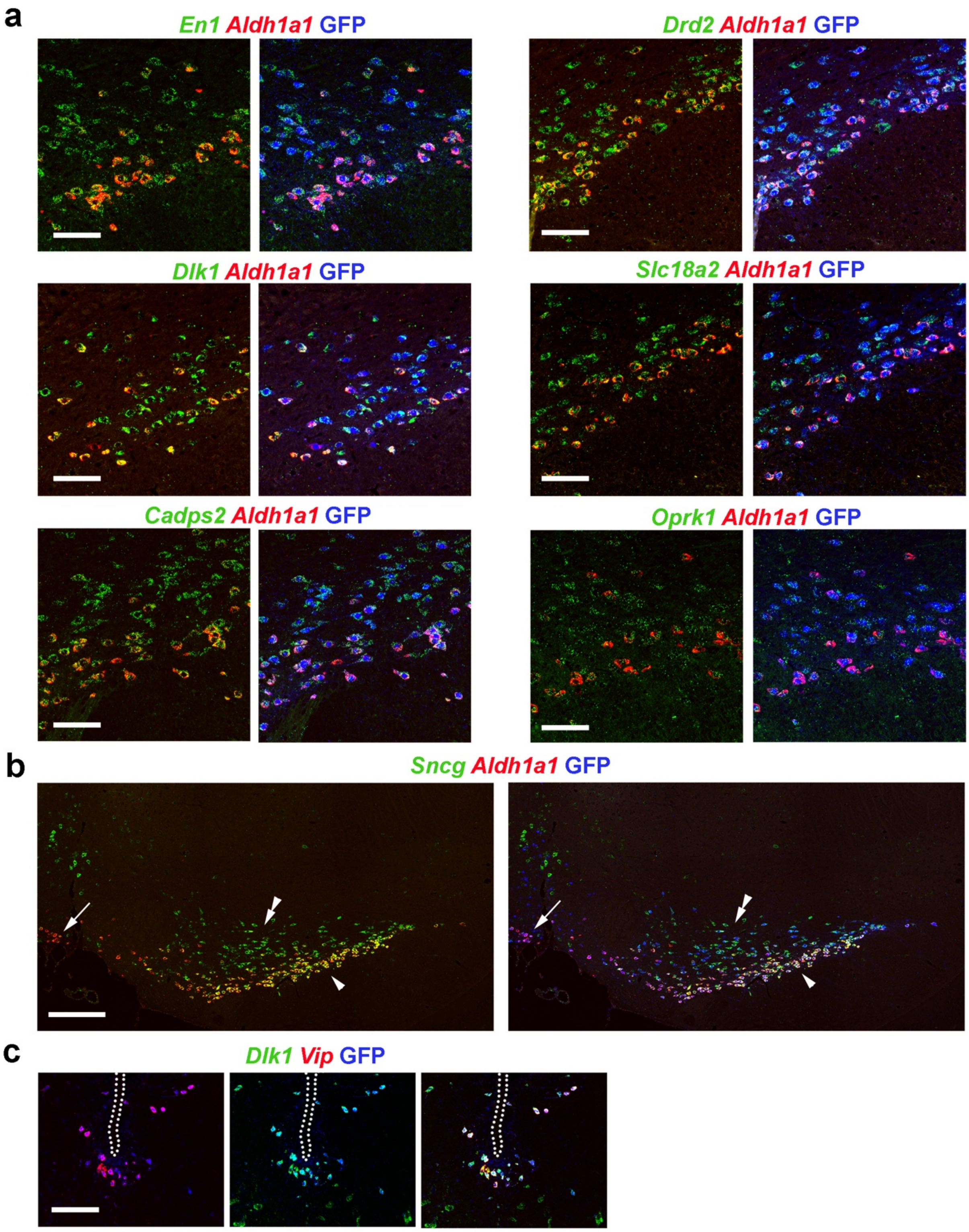

Supplementary Figure 5. Additional markers for *AT-Dat*^{high} and *VT-Dat*^{high} subgroups

a-c) Double fluorescent ISH combined with immunostaining for GFP in P90 *Pitx3*$^{eGFP/wt}$ midbrain on paraffin sections.

b) A low magnification view of *Aldh1a1* and *Sncg* expression in the ventral midbrain. *Aldh1a1* cells co-express *Sncg* in substantia nigra (arrowhead), but not in interfascicular nucleus (arrow). *Sncg Pitx3*^{eGFP} cells in parabranchial pigmented nucleus in turn lack *Aldh1a1* expression (double-arrowhead).

c) Close-up view of periaqueductal grey region showing co-expression of *Vip*, *Dlk1* and *Pitx3*^{eGFP}.

Scalebars are 100 μm in a and c, and 200 μm in b.

Sup.Figure 6

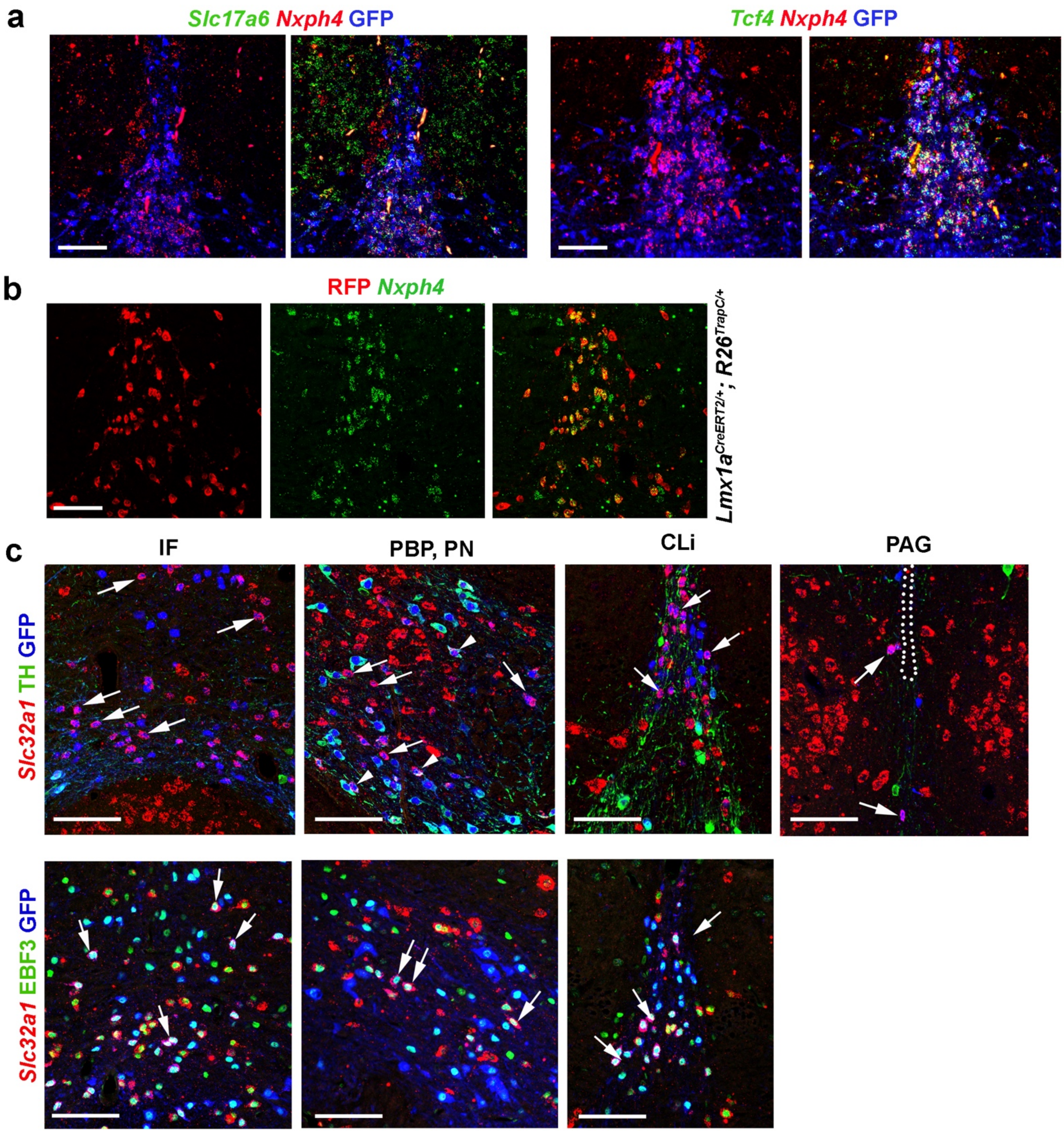

Supplementary Figure 6. Additional markers for *N-Dat*^{low}, *G-Dat*^{low} and *GT-Dat*^{low} subgroups

a) Double fluorescent ISH combined with immunostaining for GFP in P90 *Pitx3*$^{eGFP/wt}$ midbrain showing co-expression of *Nxph4* with *Slc17a6* and *Tcf4*.

b) Lineage tracing with *Lmx1a*^{CreERT2} fluorescent reporter. Tamoxifen was administered at E9.5 and samples analyzed at P90. *Nxph4* cells are labeled with the reporter showing their origin in the embryonic *Lmx1a* domain.

c) Localization of *G-Dat*^{low} and *GT-Dat*^{low} subgroups. They co-express *Slc32a1* and EBF3 in P90 *Pitx3*$^{eGFP/wt}$ midbrain. Arrows indicate *G-Dat*^{low} subgroup cells which lack detectable levels of TH protein and arrowheads cells with detectable levels of TH protein. All images are of paraffin sections. IF, interfascicular nucleus; PBP, parabrachial pigmented nucleus; PN, paranigral nucleus; CLi, caudal linear raphe; PAG, periaqueductal grey. Scalebars are 100 μm.

Sup.Figure 7

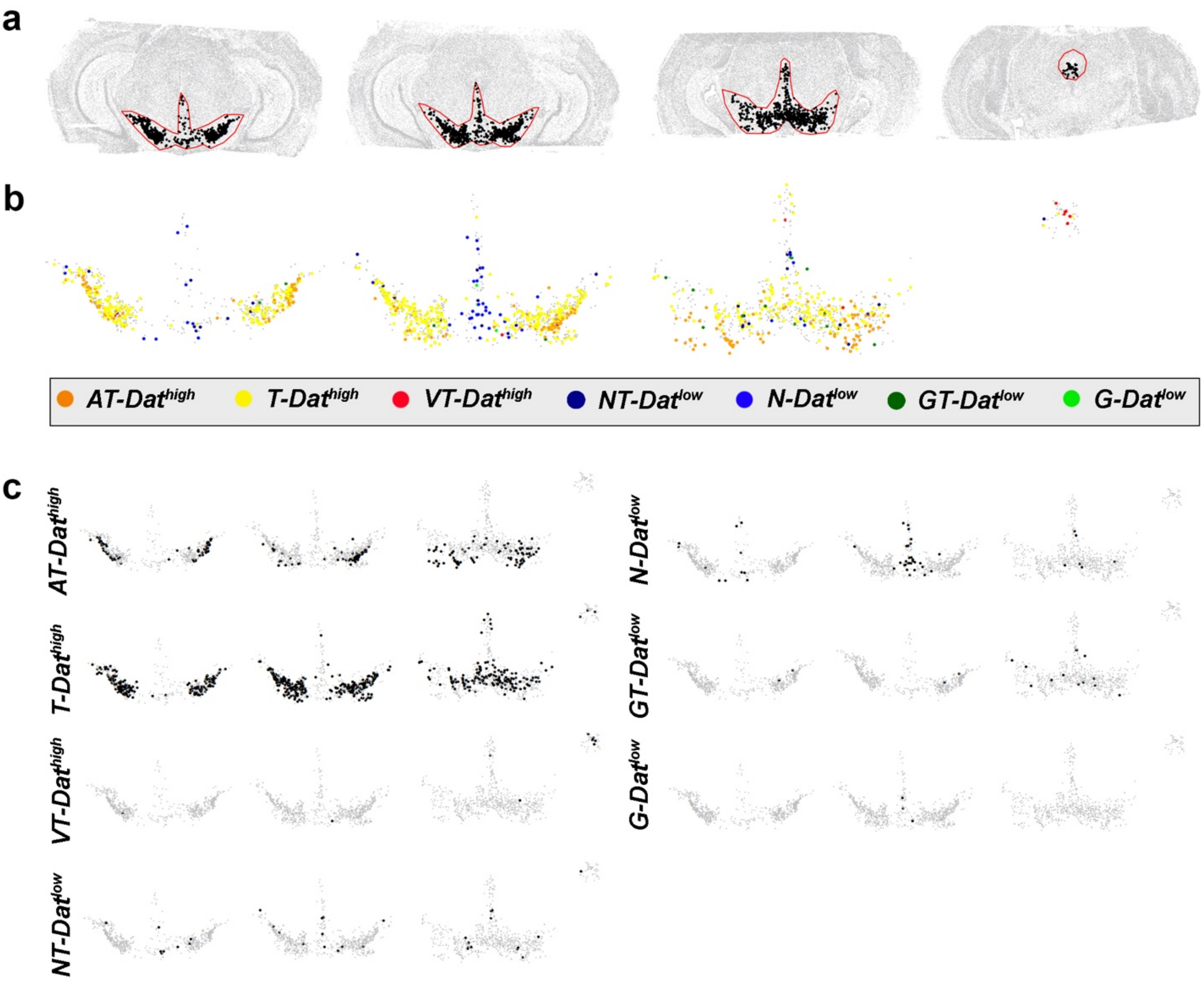

d

Dat^{high}	*Mob3b, Grik3, Zfhx3, Oprk1, Ghr, S100a10, Vwc2l, Ntf3, Slc39a4, Ntsr1, Cadps2, Sv2c*
AT-Dat^{high}	*Aldh1a1, Grp, Lpl, Nrp1, Otx2*
T-Dat^{high}	*Sncg, Npw, Unc5d*
VT-Dat^{high}	*Vip, Id4, Gipr, Pou2f2, Tdh, Wnt4, Nid1*
NT, N-Dat^{low}	*Dmrta2, Amn, Fam19a2, C1ql1, Nos1, Lypd1, Ust, Nfix, Nxph4*
GT,G-Dat^{low}	*Slc26a7, Ebf2, Gad2, Htr2c, Ghsr, Pld1, Plcl1, Crhbp, Ptprq, Wnt7b*
**All**	*Th, Pitx3, eGFP*

e

AT-Dat^{high}	23%
T-Dat^{high}	65%
VT-Dat^{high}	1%
N-Dat^{low}	5%
NT-Dat^{low}	3%
G-Dat^{low}	1%
GT-Dat^{low}	2%

Supplementary Figure 7. In situ sequencing of the *Pitx3* subgroups

a) The tissue sections of adult mouse midbrain from rostral to caudal. Each point represents an identified cell, with the region selected for analysis marked with a red polygon. The selected cells after filtering are marked with black.

b) Spatial distribution of predicted *Pitx3* subgroups among the selected cells from each section.

c) Spatial distribution of individual *Pitx3* subgroups in the adult mouse midbrain, from rostral to caudal. Black dots represent cells which are assigned to the sublineage indicated. Remaining cells are shown in light grey.

d) Genes used for mapping of the spatial distribution of the *Pitx3* subgroups in the Padlock experiment. The genes in the "Dat-high" list were common to *AT-Dat^{high}*, *T-Dat^{high}*, and *V-Dat^{high}*, and were used to identify them together with the specific genes for each of the subgroups. The three genes in "All" were used to analyse all the groups, with *Th* being present in *AT-Dat^{high}, T-Dat^{high}, VT-Dat^{high}, NT-Dat^{low}* and *GT-Dat^{low}*, and being absent in *G-Dat^{low}* and *N-Dat^{low}* subgroups. These padlock probes, chosen among the most enriched genes for each of the sublineages, were simultaneously hybridized to each tissue across the midbrain. The probes were hybridized to the target cDNA, and upon amplification, the rolling circle products were generated. These products, which will be sequenced, become visible in the sample as individual dots by fluorescence imaging (see Methods for details), generating the image. e) Proportion of *Pitx3* subgroups in the Padlock in-situ RNA-seq.

Sup. Fig. 8

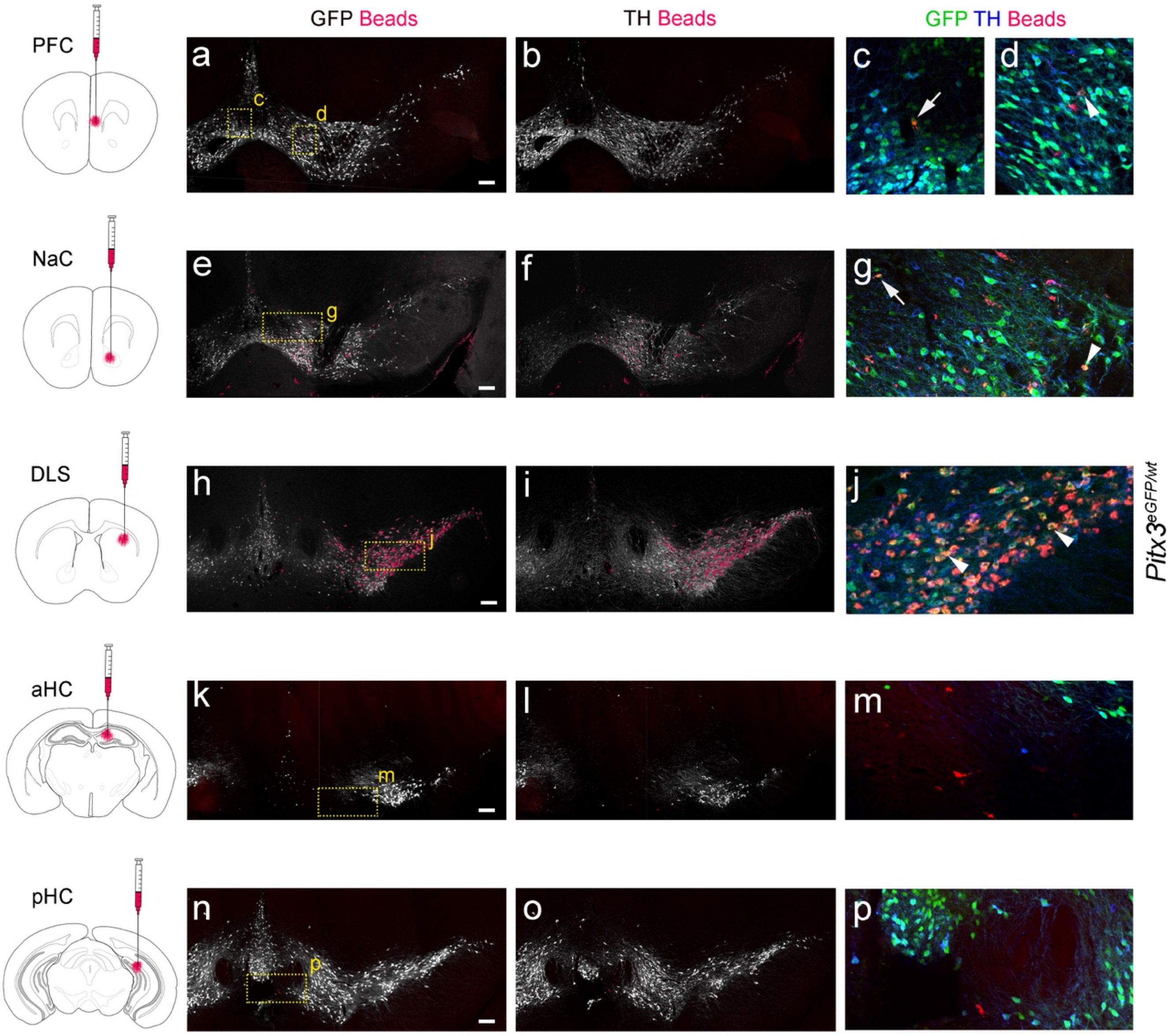

q

	PFC	NaC	DLS
DAT high	x	x	x
DAT low	x	x	

Supplementary Figure 8. Retrograde labelling of midbrain dopamine projections
Red fluorescent retrograde beads were stereotactically injected into the brain of adult *Pitx3-eGFP/^{wt}* mice, in areas indicated in the schematic figures. (a-d) Beads injected in prefrontal cortex (PFC) labelled scattered TH-negative, but Pitx3-eGFP positive, cell bodies in the medial VTA (arrow in c). Labelling was also found in few TH-expressing neurons in parabranchial pigmented nucleus (arrowhead in d), as well as in some GFP-negative cells in the same regions. (e-g) Injections in the nucleus accumbens (NaC) labelled numerous Pitx3-eGFP-positive, TH-expressing cells (arrowhead), as well as some Pitx3-eGFP-positive but TH-negative cells (arrow) in VTA. (h-j) In contrast, beads injected in dorsolateral striatum (DLS) labelled only TH-expressing SNc neurons. (k-p) No labelling of GFP-positive cells was detected with injections into anterior (aHC) or posterior (pHC) hippocampus, a possible target of VTA dopamine projections[1]. However, some labelling was seen in non-GFP-positive cells in and around VTA in these animals, as reported previously[1]. q) A table summarising the detected neuronal projections from *DAT^{high}* and *DAT^{low}* lineages. Scalebars are 100 µm.

Sup. Figure 9

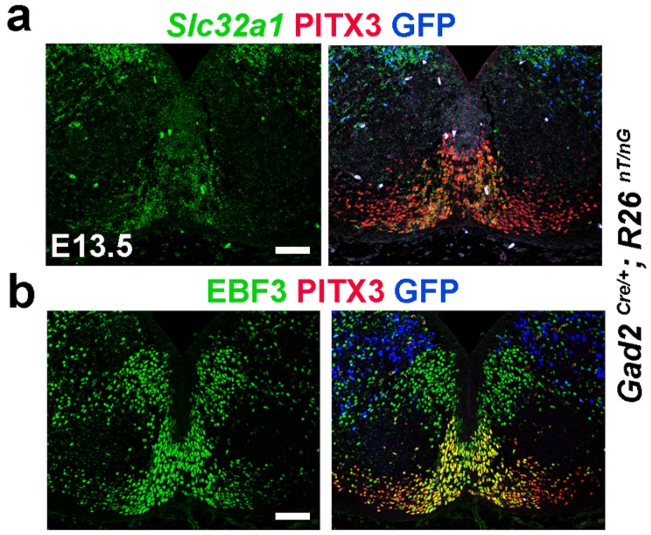

Supplementary Figure 9. Additional markers for embryonic *G-Dat*^{low} subgroup
(a,b) Co-expression of *Slc3a1* and EBF3 with PITX3 in E13.5 *Gad2*$^{Cre/+}$ fluorescent reporter, on paraffin sections. GFP detects CRE-labelled cells. Scalebars are 100 µm.

Sup.Figure 10

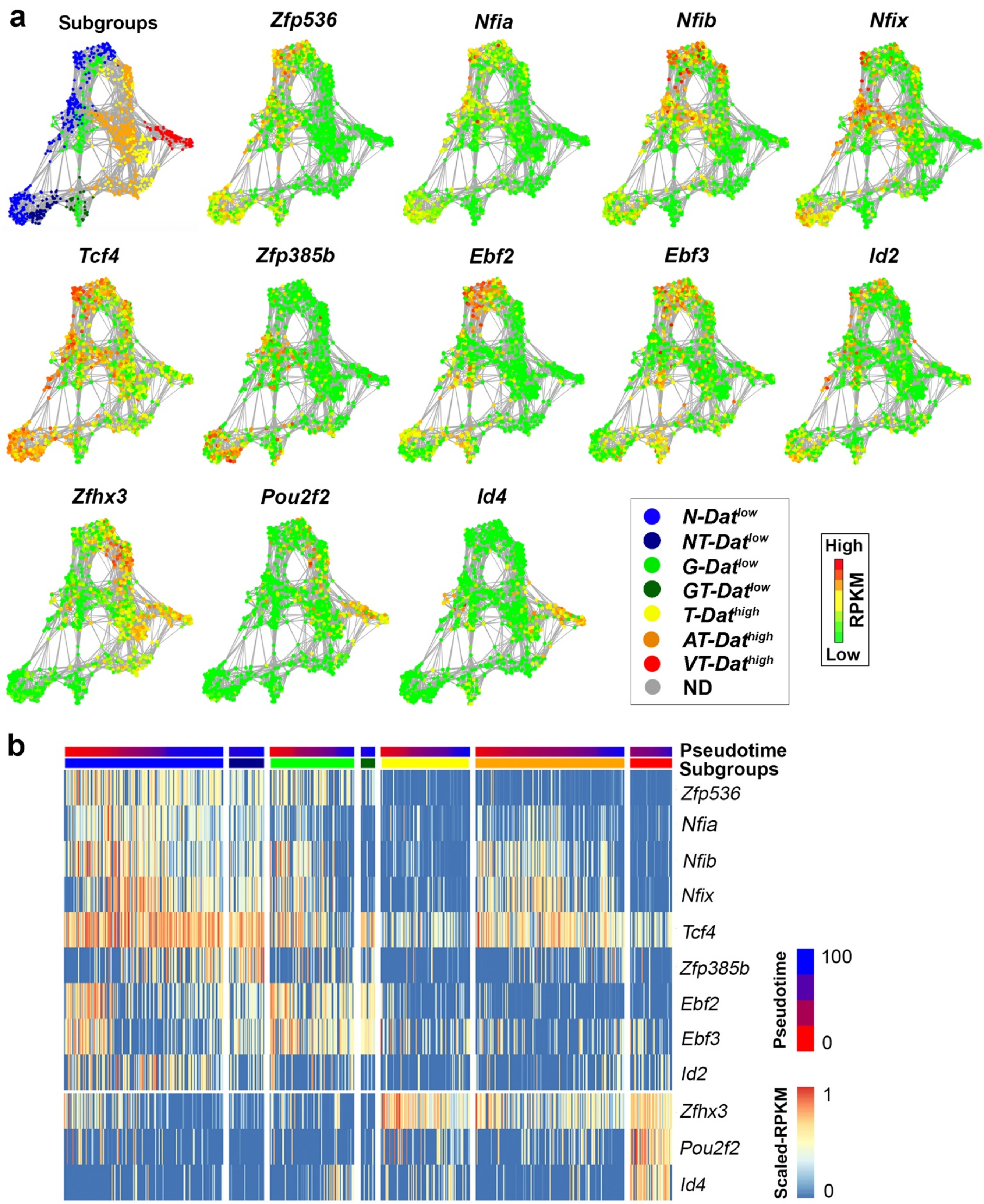

Supplementary Figure 10. Putative regulators of *Pitx3* subgroups diversification

a) Network plot visualizing the *Pitx3* subgroups and expression of several subgroup specific transcription factors. The colors indicate the level of expression (green-low, red-high).

b) Heatmap visualizing expression of the same transcription factors as in (a). The cells of each subgroup are ordered from E13.5 to P90, following pseudotime.

Sup. Figure 11

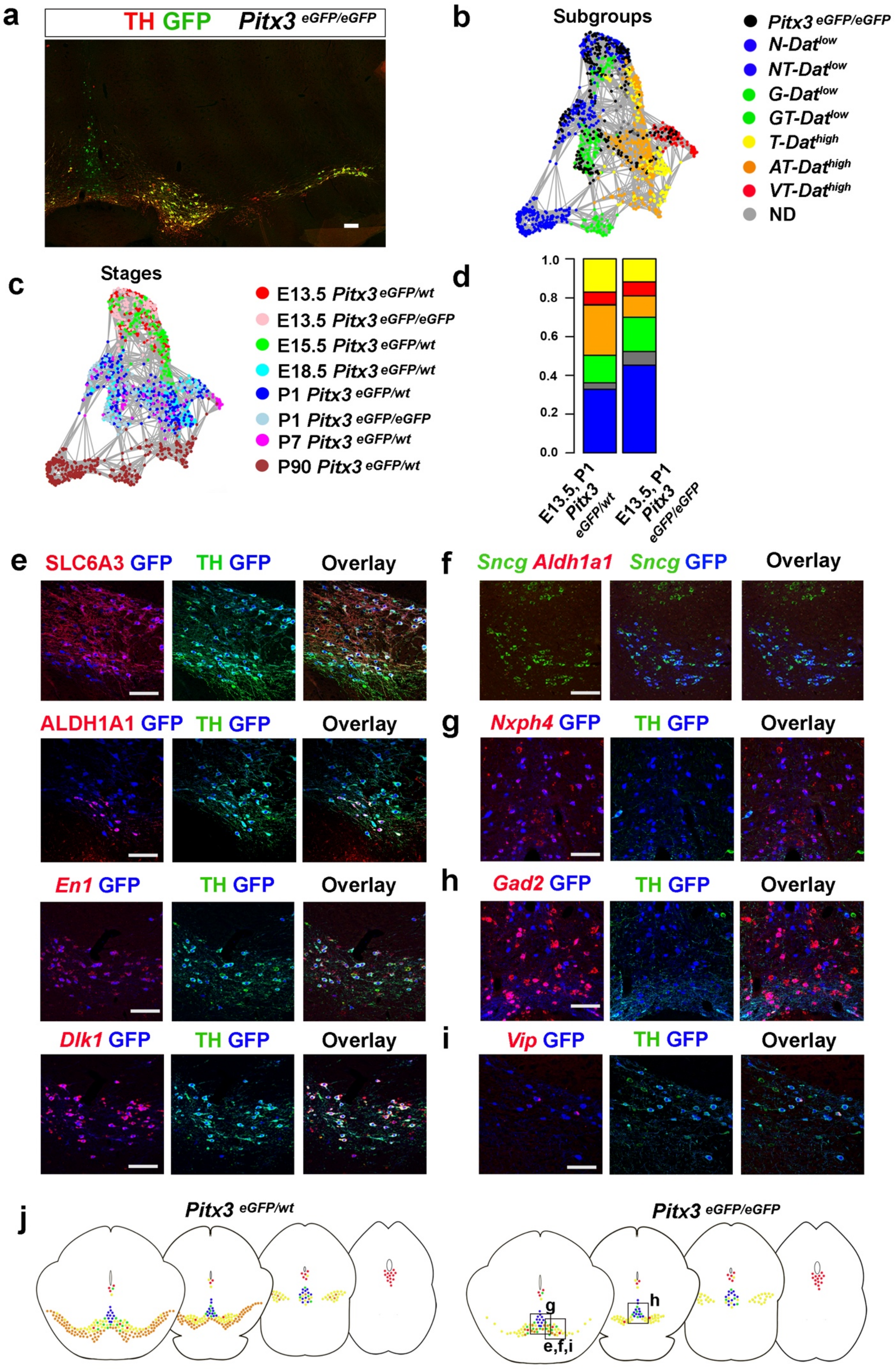

Supplementary Figure 11. Subgroups affected in *Pitx3* mutant midbrain
a) Immunostaining analysis of GFP and TH in *Pitx3*$^{eGFP/eGFP}$ mutant adult midbrain. Scalebars are 100 µm.
b) Network plot of *Pitx3* wild type and *Pitx3* mutant cells. *Pitx3*$^{eGFP/wt}$ cells are color-coded by the subgroups and *Pitx3*$^{eGFP/eGFP}$ are black. Note the lower density of *Pitx3*$^{eGFP/eGFP}$ cells in the *AT-Dat*^{high} and *T-Dat*^{high} subgroups.
c) Network plot of *Pitx3* wild type and *Pitx3* mutant cells. Cells are color-coded by the developmental stage.
d) Bar plot visualizing the distribution of the different cell subgroups among *Pitx3*$^{eGFP/wt}$ and *Pitx3*$^{eGFP/eGFP}$ cells.
e-i) Close-up views of *Pitx3* subgroups in P90 *Pitx3*$^{eGFP/eGFP}$ midbrain from the areas indicated in (j). Immunostainings for SLC6A3 and ALDH1A1 (e), for others fluorescent ISH with immunostaining for TH and GFP (f-i), all on paraffin sections. Scalebars are 100 µm.
j) Schematic representation of the *Pitx3*-expressing subgroups in the control and *Pitx3* mutant midbrain, from rostral to caudal.

Sup.Figure 12

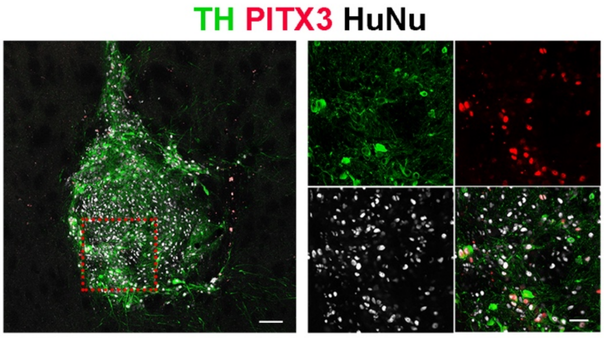

Supplementary Figure 12. Heterogeneity of human ES-mesDA graft
Immunostaining analysis of PITX3 and TH in human ES-mesDA derived graft localized in striatum. HuNu antibodies detects the human cells. Boxed area indicates the close-up images. Scalebars are 100 µm.

Sup.Figure 13

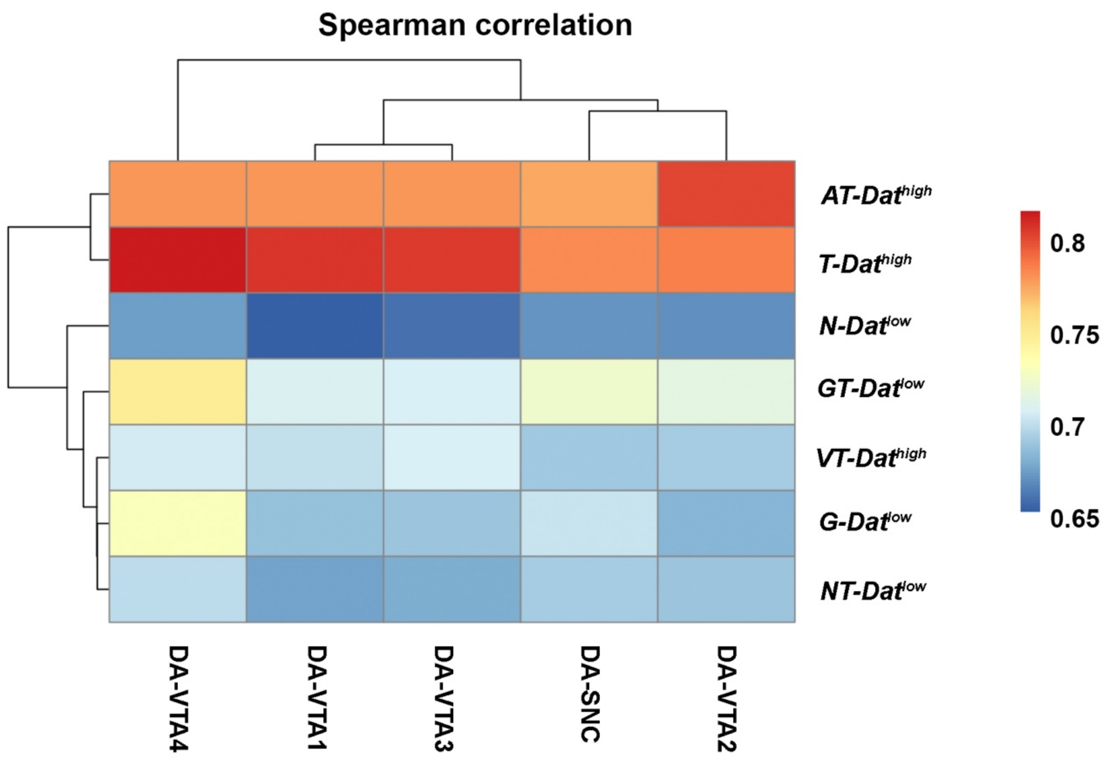

Supplementary Figure 13. Comparison to published data set
Spearman correlation of *Pitx3* subgroups (vertically) and ref. [2] (horizontally).

Supplementary Table 1. Primary and secondary antibodies

Primary antibodies					
Host	Name	Company /Ref	Product number	Dilution used	
Mouse	TH	Millipore	MAB318	1:1000	
Rabbit	TH	Pelfreeze	P40101-0	1:1000	
Rabbit	TH	Millipore	AB152	1:1000	
Rabbit	Nfib	Sigma-Aldrich	HPA003956-100UL	1:500	
Rabbit	Nfix	GeneTex	N3C3	1:400	
Rat	Slc6a3	Millipore	MAB369	1:400	
Rabbit	Aldh1a1	Abcam	ab24343	1:800	
Mouse	Ebf3	Millipore	AB10525	1:500	
Mouse	EBF3	Abnova	H00253738-M05	1:500	
Goat	GFP	Abcam	ab6673	1:800	
Guinepig	Pitx3	Self-made	N/A	1:1000	
Mouse	HuNu	Millipore	MAB1281	1:200	

Secondary antibodies					
Host	Target	Conjugate	Company	Product number	Dilution used
Donkey	Rabbit	488	Life Technologies	A21206	1:400
Donkey	Rabbit	568	Life Technologies	A10042	1:400
Donkey	Mouse	488	Life Technologies	A21202	1:400
Donkey	Mouse	568	Life Technologies	A10037	1:400
Donkey	Goat	647	Life Technologies	A21447	1:400
Donkey	Rat	Cy3	Millipore	AP189C	1:400
Donkey	Guineapig	Cy3	Jackson Immunoresearch	706-165-148	1:1000

Supplementary Table 2. cRNA probes.

Name	Source
Aldh1a1	A gift from Prof. Juha Partanen, University of Helsinki, Helsinki, Finland
Cadps2	Allen Mouse Brain Atlas, RP_051101_01_A04
Cd24a	Cloned from E14 midbrain cDNA with primers AAGCAAGAGCCAACGGTCAT and TCCAATCCACCATTGCCTGG
Dlk1	Allen Mouse Brain Atlas, RP_050725_03_A10
Drd2	Allen Mouse Brain Atlas, RP_Baylor_102735
En1	A gift from Prof. Juha Partanen, University of Helsinki, Helsinki, Finland
Gad2	Allen Mouse Brain Atlas, RP_071018_02_B07
Mkrn3	Cloned from E18 midbrain cDNA with primers CTTACCGAGGTCGCATGGTC and AAGCAAGAGCCAACGGTCAT
Nrep	Cloned from E14 midbrain cDNA with primers AGACTTCCCGTGCCTAAGGA and AGGACAGCCTCTCACACTCT
Nrsn2	Cloned from adult DA cDNA with primers TACAATGACTGGCTGGGCAC and TGATTGGGGGTTAGGGGTGA
Nxph4	Allen Mouse Brain Atlas, RP_051101_02_H07
Oprk1	Allen Mouse Brain Atlas, RP_080424_01_A07
Slc18a2	A gift from Prof. Juha Partanen, University of Helsinki, Helsinki, Finland
Slc32a1	Allen Mouse Brain Atlas, RP_051017_01_D07
Slc6a3	Allen Mouse Brain Atlas, RP_071204_02_G05
Slc17a6	A gift from Prof. Juha Partanen, University of Helsinki, Helsinki, Finland
Sncb	Cloned from adult DA cDNA with primers CGTCCTCTATGTCGGAAGCA and CACAACAAACGACGCCCG
Sncg	Allen Mouse Brain Atlas, RP_051017_01_C05
Tcf4	Allen Mouse Brain Atlas, RP_070129_01_A08
Vip	Allen Mouse Brain Atlas, RP_070116_02_E09

Supplementary References

[1] Gasbarri, A., Verney, C., Innocenzi, R., Campana, E. & Pacitti, C. Mesolimbic dopaminergic neurons innervating the hippocampal formation in the rat: a combined retrograde tracing and immunohistochemical study. *Brain Res* **668,** 71–79 (1994).

[2] La Manno, G. *et al.* Molecular Diversity of Midbrain Development in Mouse, Human, and Stem Cells. *Cell* **167,** 566–580.e19 (2016).
