# Supplementary material for: Single-cell RNA sequencing reveals midbrain dopamine neuron diversity emerging during mouse brain development
Source: Nat Commun. 2019 Feb 4;10:581. doi: 10.1038/s41467-019-08453-1 (PMC6362095; doi:10.1038/s41467-019-08453-1)
Supplement: Supplementary file 3 — Description of Additional Supplementary Information [file 41467_2019_8453_MOESM3_ESM.pdf]

Description of Additional Supplementary Files

File Name: Supplementary Data 1
Description: Maturation gene list

File Name: Supplementary Data 2
Description: Subgroups gene list

File Name: Supplementary Data 3
Description: Padlock probe list

File Name: Supplementary Data 4
Description: Expression signal of padlock probes list
