# Supplementary material for: Single-cell RNA sequencing reveals midbrain dopamine neuron diversity emerging during mouse brain development
Source: Nat Commun. 2019 Feb 4;10:581. doi: 10.1038/s41467-019-08453-1 (PMC6362095; doi:10.1038/s41467-019-08453-1)
Supplement: Supplementary file 8 — Reporting Summary [file 41467_2019_8453_MOESM8_ESM.pdf]

nature research

Corresponding author(s): Thomas Perlmann

Last updated by author(s): Jan 10, 2019

Reporting Summary

Nature Research wishes to improve the reproducibility of the work that we publish. This form provides structure for consistency and transparency in reporting. For further information on Nature Research policies, see Authors & Referees and the Editorial Policy Checklist.

Statistics

For all statistical analyses, confirm that the following items are present in the figure legend, table legend, main text, or Methods section.

n/a	Confirmed	
☒	☐	The exact sample size (n) for each experimental group/condition, given as a discrete number and unit of measurement
☒	☐	A statement on whether measurements were taken from distinct samples or whether the same sample was measured repeatedly
☒	☐	The statistical test(s) used AND whether they are one- or two-sided<br>*Only common tests should be described solely by name; describe more complex techniques in the Methods section.*
☒	☐	A description of all covariates tested
☒	☐	A description of any assumptions or corrections, such as tests of normality and adjustment for multiple comparisons
☒	☐	A full description of the statistical parameters including central tendency (e.g. means) or other basic estimates (e.g. regression coefficient) AND variation (e.g. standard deviation) or associated estimates of uncertainty (e.g. confidence intervals)
☒	☐	For null hypothesis testing, the test statistic (e.g. F, t, r) with confidence intervals, effect sizes, degrees of freedom and P value noted<br>*Give P values as exact values whenever suitable.*
☒	☐	For Bayesian analysis, information on the choice of priors and Markov chain Monte Carlo settings
☒	☐	For hierarchical and complex designs, identification of the appropriate level for tests and full reporting of outcomes
☒	☐	Estimates of effect sizes (e.g. Cohen's d, Pearson's r), indicating how they were calculated

Our web collection on statistics for biologists contains articles on many of the points above.

Software and code

Policy information about availability of computer code

Data collection	Single cell sequencing data was collected using Illumina HiSeq 2000. mRNA ISH, IHC samples and in situ RNA sequencing were imaged using Zeiss ZEN software. Fluorescences intensities for in situ RNA sequencing were extracted using a custom-made Cellprofiler 2.2.1 pipline.
Data analysis	Single cell alignment was analyzed using Star v2.3.0. Variable gene sets were analyzed using t-SNE. Networks were created using igraph. Clustering was done with Infomap. SAMseq was used to define maturation and subpopulation-specific genes. Gene changes along pseudotime were analyzed with Monocle and gene cluster analyzed for biological functions using Ingenuity Pathway Analysis program. mRNA ISH and ICH samples were processed using Adobe Photoshop CC 2017. Signal decoding of in situ RNA sequencing was done using Matlab 2018a. Cluster prediction for each in situ cell was analyzed using randomForest R.

For manuscripts utilizing custom algorithms or software that are central to the research but not yet described in published literature, software must be made available to editors/reviewers. We strongly encourage code deposition in a community repository (e.g. GitHub). See the Nature Research guidelines for submitting code & software for further information.

Data

Policy information about availability of data

All manuscripts must include a data availability statement. This statement should provide the following information, where applicable:

- Accession codes, unique identifiers, or web links for publicly available datasets
- A list of figures that have associated raw data
- A description of any restrictions on data availability

Data supporting the findings in this study are within this manuscript, on the website http://perlmannlab.org, or available from the corresponding authors upon request. The RNA-seq data have been submitted to the GEO database under the accession code GSE116138.

Field-specific reporting

Please select the one below that is the best fit for your research. If you are not sure, read the appropriate sections before making your selection.

☒ Life sciences ☐ Behavioural & social sciences ☐ Ecological, evolutionary & environmental sciences

For a reference copy of the document with all sections, see nature.com/documents/nr-reporting-summary-flat.pdf

Life sciences study design

All studies must disclose on these points even when the disclosure is negative.

Sample size	A minimum of 3 animals per embryonic or postnatal stage and for each genotype were analyzed in each experiment. No statistical methods were used to predetermine sample size.
Data exclusions	For the single cells we have used the following cell exclusion criteria for E13.5, E15.5, E18.5, P1, P7 cells : > 17.1% uniquely mapping reads, < 66% fraction mismatches, > 62% exon mapping reads, < 7.8% 3'mapping, at least 15% of all genes detected, > 100000 normalization reads and for P90 cells > 21% uniquely mapping reads, < 80% fraction mismatches, > 61% exon mapping reads, < 8.8% 3'mapping, at least 3.4% of all genes detected, > 100000 normalization reads. From 1699 sequenced cells 1562 passed the quality control. Another 167 cells were excluded from analysis since they showed high Olig1 expression, represSenting oligodendrocytes.
Replication	Sequencing comprised one data set without replications. All the validations were replicated at least once and the replications confirmed the original results.
Randomization	Randomization of samples were not applicable in this paper.
Blinding	Blinding of samples were not applicable in this paper.

Reporting for specific materials, systems and methods

We require information from authors about some types of materials, experimental systems and methods used in many studies. Here, indicate whether each material, system or method listed is relevant to your study. If you are not sure if a list item applies to your research, read the appropriate section before selecting a response.

Materials & experimental systems

n/a	Involved in the study	
☐	☒	Antibodies
☒	☐	Eukaryotic cell lines
☒	☐	Palaeontology
☐	☒	Animals and other organisms
☐	☒	Human research participants
☒	☐	Clinical data

Methods

n/a	Involved in the study	
☒	☐	ChIP-seq
☐	☒	Flow cytometry
☒	☐	MRI-based neuroimaging

Antibodies

Antibodies used	Please see supplementary table 3.
Validation	Relevant information is available in the Supplementary table 3

Animals and other organisms

Policy information about studies involving animals; ARRIVE guidelines recommended for reporting animal research

Laboratory animals	Mosue lines used are described in detail under methods.
Wild animals	No wild animals were used.
Field-collected samples	No samples collected from the field.
Ethics oversight	All experimental procedures followed the guidelines and recommendations of Swedish animal protection legislation and were approved by Stockholm North Animal Ethics board.Human fetal tissue was obtained from legally terminated embryos with approval of the Swedish National Board of Health and Welfare in accordance with existing guidelines including informed consent from women seeking elective abortions.

Note that full information on the approval of the study protocol must also be provided in the manuscript.

Human research participants

Policy information about studies involving human research participants

Population characteristics	No human subjects were included. Embryonic tissue collected according to ethics board permit (see below).
Recruitment	Human fetal tissue was obtained from legally terminated embryos with informed consent from women seeking elective abortion
Ethics oversight	Swedish National Board of Health and Welfare

Note that full information on the approval of the study protocol must also be provided in the manuscript.

Flow Cytometry

Plots

Confirm that:

- [x] The axis labels state the marker and fluorochrome used (e.g. CD4-FITC).
- [x] The axis scales are clearly visible. Include numbers along axes only for bottom left plot of group (a 'group' is an analysis of identical markers).
- [x] All plots are contour plots with outliers or pseudocolor plots.
- [ ] A numerical value for number of cells or percentage (with statistics) is provided.

Methodology

Sample preparation	Ventral midbrain tissue was dissected from Pitx3eGFP reporter mice and dissociated into a single cell suspension as using the papain kit (Worthington).
Instrument	Cells were FACS sorted using a BD FACSAria III Cell Sorter
Software	FlowJo v10.4.2
Cell population abundance	Purity of samples is assessed from the single cell transcriptomes .
Gating strategy	Ventral midbrain cells were sorted using forward scatter area/side scatter area (FSC-A/ SSC-A), side scatter height/side scatter width (SSC-H/ SSC-W), followed by detection of fluorescent events FITC-A/PE-A. GFP was excited with a 488 nm laser. A figure describing the gating strategy is shown in Supplementary Figure 1d.

- [x] Tick this box to confirm that a figure exemplifying the gating strategy is provided in the Supplementary Information.
